# DNA Methylation Is Correlated with Oxidative Stress in Myelodysplastic Syndrome—Relevance as Complementary Prognostic Biomarkers

**DOI:** 10.3390/cancers13133138

**Published:** 2021-06-23

**Authors:** Ana Cristina Gonçalves, Raquel Alves, Inês Baldeiras, Bárbara Marques, Bárbara Oliveiros, Amélia Pereira, José Manuel Nascimento Costa, Emília Cortesão, Luisa Mota Vieira, Ana Bela Sarmento Ribeiro

**Affiliations:** 1 Laboratory of Oncobiology and Hematology and University Clinic of Hematology, Faculty of Medicine (FMUC), University of Coimbra, 3000-370 Coimbra, Portugal; acgoncalves@fmed.uc.pt (A.C.G.); rakel.silva.alves@gmail.com (R.A.); barbara_marques3@hotmail.com (B.M.); ecortesao@sapo.pt (E.C.); 2 Institute for Clinical and Biomedical Research (iCBR)—Group of Environment, Genetics and Oncobiology (CIMAGO), FMUC, University of Coimbra, 3000-370 Coimbra, Portugal; boliveiros@fmed.uc.pt (B.O.); ameliafpereira@gmail.com (A.P.); jmnc@gmail.com (J.M.N.C.); 3 Center for Innovative Biomedicine and Biotechnology (CIBB), University of Coimbra, 3000-370 Coimbra, Portugal; 4 Clinical Academic Center of Coimbra (CACC), 3004-561 Coimbra, Portugal; 5 Faculty of Medicine (FMUC), University of Coimbra, 3000-370 Coimbra, Portugal; ines.baldeiras@sapo.pt; 6 Center for Neuroscience and Cell Biology, University of Coimbra, 3000-370 Coimbra, Portugal; 7 Clinical Hematology Department, Centro Hospitalar e Universitário de Coimbra (CHUC), 3004-561 Coimbra, Portugal; 8 Medicine Department, Hospital da Luz, 3020-479 Coimbra, Portugal; 9 University Clinic of Oncology, Faculty of Medicine (FMUC), University of Coimbra, 3000-370 Coimbra, Portugal; 10 Molecular Genetics and Pathology Unit, Hospital do Divino Espirito Santo de Ponta Delgada EPER, São Miguel Island, 9500-370 Azores, Portugal; 11 Faculty of Sciences, BioISI—Biosystems & Integrative Sciences Institute, University of Lisbon, 1649-004 Lisbon, Portugal; 12 Azores Genetics Research Group, Instituto Gulbenkian de Ciência, 2780-156 Oeiras, Portugal

**Keywords:** myelodysplastic syndrome, acute myeloid leukemia, oxidative stress, DNA methylation, blood biomarkers, survival, progression, prognosis

## Abstract

**Simple Summary:**

Myelodysplastic syndrome (MDS) is a hematological malignancy with a high propensity to evolve to acute myeloid leukemia. Oxidative stress and abnormal DNA methylation are important in this neoplasia’s development and progression. We investigate whether oxidative stress parameters were correlated with localized and global DNA methylations in the peripheral blood of patients with MDS. We found that oxidative stress was positively correlated with DNA methylation and associated with worse overall survival. Biologically, these facts suggest a relationship between oxidative stress and DNA methylation, two common pathogenic mechanisms involved in MDS. Clinically, our findings can improve an MDS patient’s management if used as complementary prognostic biomarkers.

**Abstract:**

Oxidative stress and abnormal DNA methylation have been implicated in cancer, including myelodysplastic syndromes (MDSs). This fact leads us to investigate whether oxidative stress is correlated with localized and global DNA methylations in the peripheral blood of MDS patients. Sixty-six MDS patients and 26 healthy individuals were analyzed. Several oxidative stress and macromolecule damage parameters were analyzed. Localized (gene promotor) and global DNA methylations (5-mC and 5-hmC levels; LINE-1 methylation) were assessed. MDS patients had lower levels of reduced glutathione and total antioxidant status (TAS) and higher levels of peroxides, nitric oxide, peroxides/TAS, and 8-hydroxy-2-deoxyguanosine compared with controls. These patients had higher 5-mC levels and lower 5-hmC/5-mC ratio and LINE-1 methylation and increased methylation frequency of at least one methylated gene. Peroxide levels and peroxide/TAS ratio were higher in patients with methylated genes than those without methylation and negatively correlated with LINE-1 methylation and positively with 5-mC levels. The 5-hmC/5-mC ratio was significantly associated with progression to acute leukemia and peroxide/TAS ratio with overall survival. This study points to a relationship between oxidative stress and DNA methylation, two common pathogenic mechanisms involved in MDS, and suggests the relevance of 5-hmC/5-mC and peroxide/TAS ratios as complementary prognostic biomarkers.

## 1. Introduction

Myelodysplastic syndromes (MDSs) are a heterogeneous group of clonal stem cell disorders characterized by dysplasia, impaired differentiation, and inefficient hematopoiesis, leading to progressive peripheral cytopenias with several degrees. Patients with these diseases show a high propensity for transformation into acute myeloid leukemia (AML) [1,2,3]. The clinical course of MDS is highly heterogeneous, ranging from mild symptoms over several years to a quickly progressive disease that progresses into AML [4,5]. It is recognized that multiple genetic and epigenetic modifications, which change gene expression, are required for the development of MDS [4]. About 50% of MDS cases have cytogenetic abnormalities, with the majority of these abnormalities being unbalanced alterations, which result in copy number abnormalities, such as gain or loss of chromosomal material [6,7,8].

Reactive oxygen species (ROS) are considered an important player in the initiation and progression of hematological malignancies [9]. They can have both beneficial and deleterious effects [10,11]. In the case of imbalance in redox homeostasis, ROS levels overwhelm cellular antioxidant defenses, and oxidative stress is established [9,11]. Several biological processes—namely, those involved in activating signaling pathways, such as proliferation, differentiation, and cell death—are dependent on appropriate intracellular ROS levels [10,12]. An increase in ROS levels with a decrease in GSH content has already been observed in blood cells from MDS patients [13,14]. High levels of ROS may contribute to cancer development through both genetic and epigenetic mechanisms [15]. At an epigenetic level, both DNA hypermethylation and hypomethylation can be induced by ROS [15,16,17,18].

In MDS, two different epigenetic alterations occur: aberrant DNA methylation and mutations in epigenetic regulator genes [19,20,21,22]. The hypermethylation of genes crucial to cell survival, differentiation, and proliferation, such as the *CDKN2B* (*P15*), *CDKN2A* (*P16*), *DAPK*, and *MGMT* genes, is observed in these myeloid malignancies [19,20]. Furthermore, MDS patients have genomic hypermethylation, and this hypermethylation increases during MDS progression to AML [23,24]. Additionally, genomic DNA methylation profiles identify clinically relevant MDS subtypes [23]. DNA hypomethylation of the cancer genome occurs in repetitive sequences, namely, in long interspersed nuclear element-1 (LINE-1) [25,26]. On the other hand, several genes involved in the regulation of DNA methylation, such as *DNMT3A*, *TET2*, *IDH1*, *IDH2*, *EZH2*, and *ASXL1*, are mutated in MDS patients [19,22].

Despite the advances in understanding myeloid malignancies’ pathogenesis, the link between these two common molecular mechanisms—oxidative stress and abnormal methylation—remains poorly understood. In a previous pilot study involving 27 MDS patients, we first demonstrated that MDS patients with methylated *P15* and *P16* gene promoters had high levels of intracellular peroxides and superoxide anion, as well as those with high ratios of peroxides/reduced glutathione (GSH) and superoxide/GSH [27]. Peripheral blood (PB) collection is more convenient for patients than bone marrow (BM) aspiration due to its less invasive nature. In this context, we first analyzed the concordance between the DNA methylation patterns in BM and PB samples in MDS patients and found a good correlation between gene methylation patterns [28]. The oxidative stress statuses (intracellular levels of peroxides, GSH, and peroxide/GSH ratio) were also compared between PB and BM samples from MDS patients (*n* = 10) and controls (*n* = 8) previously enrolled in our pilot study [27]. This analysis also revealed a good correlation between oxidative stress parameters analyzed in PB and BM samples [29]. The primary goal of the present work was to investigate whether oxidative stress is correlated with localized and global DNA methylations in MDS patients. In addition, we aimed to analyze the usefulness of these parameters as biomarkers for the diagnosis and prognosis of MDS. We found that peroxide levels and peroxide/total antioxidant status (TAS) ratio were higher in patients with methylated genes and negatively correlated with LINE-1 methylation and positively with 5-mC levels. Furthermore, the 5-hmC/5-mC ratio was significantly associated with progression to acute leukemia, and the peroxide/TAS ratio with overall survival.

## 2. Materials and Methods

### 2.1. Study Population

In the present study, 66 patients with MDS at diagnosis and 26 controls (healthy individuals without hematological (namely, any form of anemia and immune thrombocytopenia), oncologic (any malignancy), and oxidative-stress-related diseases (namely, inflammatory disease, diabetes, neurodegenerative disease)), were enrolled from October 2012 to March 2014. The exclusion criteria were: patients with a history of another primary malignancy, other concomitant malignancy, inflammatory disease, and neurodegenerative disease. MDS patients were diagnosed according to the World Health Organization 2016 classification of myeloid neoplasms [30] in the following subtypes: MDS with single lineage dysplasia (MDS-SLD), MDS with multilineage dysplasia (MDS-MD), MDS with ring sideroblasts (MDS-RS), and MDS with excess blasts (MDS-EB). Patients were stratified according to the Revised International Prognostic Scoring System (IPSS-R). Since in clinical practice, a cut-off IPSS-R score of 3.5 allows clinicians to distinguish between patients with lower-risk MDS (score ≤3.5) and those with higher-risk MDS (score >3.5) [4], MDS patients were stratified as lower- and higher-risk patients. Biodemographic (age and gender) and clinical data, when available, were obtained from medical records.

The Ethics Committee of the Faculty of Medicine of the University of Coimbra (Coimbra, Portugal) approved the research procedures, and the study was conducted following the Declaration of Helsinki. Prior to enrollment, participants provided their informed consent for participation. The international ethical guidelines of confidentiality, anonymity of personal data, and abandonment option in case of expressed will were followed.

### 2.2. Sample Preparation

At diagnosis, peripheral blood samples were collected, after fasting, into sodium heparin (oxidative stress studies) and EDTA tubes (methylation studies). Samples for oxidative stress evaluation were immediately centrifuged; plasma and red blood cells (with a concentration of hemoglobin adjusted at 100 g/L) were stored frozen at −20 °C until analysis, as previously described [31]. For normalization of some oxidative stress parameters, total plasma protein and cholesterol were measured.

### 2.3. Uric Acid Determinations

Plasmatic levels of uric acid were determined by a colorimetric method [32]. This method was based on the reduction of uric acid by the enzyme uricase, which releases hydrogen peroxide and forms a chromogenic compound, which was then spectrophotometrically evaluated at 550 nm.

### 2.4. Vitamin A and E Measurements

The assessment of plasmatic levels of vitamins A (vit A) and E (vit E) was initiated by lipid extraction from plasma samples. Next, vitamins were quantified by high-performance liquid chromatography (HPLC) using the analytic column Spherisorb ODS1 5 μm (250 × 4.6 mm), eluted at 2.5 mL/min with a water solution of methanol (90%), at 45 °C, with spectrophotometric detection (Gilson, Middleton, WI, USA) at 340 nm (for vit A) or 295 nm (for vit E) [33,34]. The levels of vitamin E in red blood cells were extracted in n-hexane and quantified by reverse-phase HPLC [33,34] using the analytic column Spherisorb S10w (250 × 4.6 mm), eluted at 1.5 mL/min with n-hexane modified with 0.9% of methanol, and detected by spectrophotometry at 287 nm (Gilson). 

### 2.5. Reduced Glutathione Quantification

GSH in red blood cells was also evaluated by HPLC with fluorimetric detection (excitation at 385 nm and emission at 515 nm) using the Immundiagnostik kit (Immundiagnostik AG, Bensheim, Germany), as described by the manufacturer.

### 2.6. Total Antioxidant Status Evaluation

Total antioxidant status (TAS) was evaluated by a chromogenic method (Randox Laboratories (Crumlin, UK)) based on the plasma capacity to inhibit the formation of the ABTS+ radical cation (2,2′-azino-di [3-etilbenzotiazolin sulfonate]) and detected at 600 nm, as described by the manufacturer.

### 2.7. Antioxidant Enzyme Activity Determination

Erythrocyte glutathione peroxidase (GPX) was evaluated by spectrophotometry using an indirect determination method and tert butyl hydroperoxide as a substrate [35]. The oxidized glutathione formation was monitored through the quantification of reduced nicotinamide adenine dinucleotide phosphate (NADPH) oxidation at 340 nm in a thermostatized spectrophotometer, UVIKON 933 UV/Visible. Erythrocyte glutathione reductase (GR) was evaluated by spectrophotometry at 340 nm [36] using GSSG as a substrate and monitoring its reduction to GSH through the quantification of NADPH oxidation at 37 °C in a spectrophotometer, UVIKON 933 UV/Visible.

### 2.8. Lipid Peroxidation Measurements

Levels of lipid peroxidation in plasma and red blood cells were assessed by the formation of thiobarbituric acid (TBA) adducts of malondialdehyde (MDA), separated by HPLC (Gilson (Middleton, WI, USA)), and quantified fluorimetrically using the ClinRep complete kit (RECIPE, Munich, Germany), as described by the manufacturer. Briefly, 100 µL blank, standard, control, and patient samples were first derivatized at 100 °C for 60 min in a glass light-protected vial. After cooling, samples were neutralized, precipitated, and centrifuged at 10,000× *g* for 5 min. Finally, 20 µL of the supernatants were injected into the HPLC, and the MDA adducts were determined fluorimetrically (excitation at 515 nm and emission at 553 nm; FP-2020/2025, Jasco, Tokyo, Japan).

### 2.9. Plasmatic Nitric Oxide Quantification 

The plasmatic levels of nitric oxide (NO) were determined by a photometric method (Roche Diagnostics GmbH, Mannheim, Germany) via its oxidation products, nitrite and nitrate [37]. First, the nitrate present in the ultrafiltrated plasma was reduced to nitrite, which then reacted with sulfanilamide and N (1 naphthyl) ethylenediamine dihydrochloride to give a red-violet diazo dye, detected by spectrophotometry at 550 nm.

### 2.10. Plasmatic Peroxide Quantification 

The plasmatic levels of peroxide were measured by a colorimetric method (Thermo Scientific Pierce Quantitative Peroxide Assay Kit, lipid-compatible formulation, Life Technologies (Carlsbad, CA, USA)), based on the oxidation of ferrous to ferric in the presence of xylenol orange. Plasmatic peroxides were detected by spectrophotometry at 595 nm and determined by comparison with a hydrogen peroxide standard curve, as described by the manufacturer, in a Synergy^TM^ multimode microplate reader (BioTek Instruments (Winooski, VT, USA).

### 2.11. Plasmatic 8-Hydroxy-2-Deoxyguanosine Quantification 

The plasmatic 8-OHdG levels were measured using a competitive quantitative ELISA Kit (8-hydroxy-2-deoxyguanosine ELISA Kit, Abcam (Cambridge, UK)), according to the manufacturer’s instructions. The assay is based on the competition between 8-OHdG and an 8-OHdG acetylcholinesterase conjugate for a limited amount of 8-OHdG monoclonal antibody. The colorimetric intensity was determined spectrophotometrically in a Synergy^TM^ multimode microplate reader, and its value was inversely proportional to the amount of free 8-OHdG in plasma.

### 2.12. Global DNA Methylation Analysis

Global methylation and hydroxymethylation were determined in DNA extracted from leukocytes obtained from peripheral blood collected into EDTA tubes by specific ELISA assays (5-methylcytosine DNA ELISA Kit and 5-hydroxymethylcytosine DNA ELISA Kit, respectively, Enzo, Farmingdale, NY, USA), according to the manufacturer’s protocol. Genomic DNA was extracted from whole blood, as previously described by Bartlett and Stirling [38]. DNA was quantified using a NanoDrop ND-1000 spectrophotometer (NanoDrop Technologies (Wilmington, DE, USA)). These assays use monoclonal antibodies against 5-methylcytosine (5-mC) and 5-hydroxymethylcytosine (5-hmC) to obtain the percentage of 5-mC and 5-hmC in total DNA. Global methylation was also assessed by methylation analysis of LINE-1 repetitive elements using HRM-PCR as previously described [39]. 

### 2.13. Methylation Pattern of Tumor Suppressor Genes

The methylation statuses of the tumor suppressor genes *P15*, *P16*, *TP53*, *MGMT*, *DAPK*, and *KEAP1* were carried out in DNA extracted from peripheral blood leukocytes by methylation-specific PCR (MSP), as previously described by others [40,41,42,43]. Each assay was validated by the amplification of unmethylated and methylated universal DNA controls (EpiTect PCR Control DNA Set, Qiagen (Hilden, Germany)) and a no template control (NTC). PCR products were resolved on 3% agarose gel stained with ethidium bromide and visualized under UV illumination. 

### 2.14. Statistical Analysis

Statistical analysis was performed using SPSS (version 27.0), and graphics were constructed through GraphPad Prism (version 6.0), Orange (version 3.28.0), and R (version 4.0.3). Continuous variables were expressed as median (quartile 1—quartile 3) unless otherwise specified, and categorical variables as numbers and percentages. Since lipids affect the concentration of fat-soluble vitamins, to minimize the effects of plasma lipid content in vitamin A and E levels, these vitamins were expressed in relation to cholesterol content. All statistical analyses were two-sided, and a *p* < 0.05 was considered statistically significant. Normality was assessed by the Kolmogorov–Smirnov test. Student’s *t*-test or ANOVA with Bonferroni post hoc test was performed for normally distributed continuous variables. When continuous variables did not show normal distribution, the Mann–Whitney U or Kruskal–Wallis tests were used. Spearman or Pearson correlation coefficients determined the association between continuous variables. The methylation frequency between groups was compared using the chi-square test. Logistic regression was performed to establish the factors associated with MDS and correlate oxidative stress factors with DNA methylation. Factors that showed a significant association in the univariate analysis were included in the multivariate logistic regression to determine the independent variables associated. Finally, receiver operating characteristic (ROC) curves were performed to evaluate the accuracy of significant parameters as diagnostic biomarkers of MDS. The optimal cut-off point was determined using Youden’s J index. Survival analysis was performed by the Kaplan–Meier method using the cut-off points obtained from the ROC curves constructed to predict death and progression. Differences in survival were tested through log-rank statistics.

## 3. Results

### 3.1. Biodemographic and Clinical Characteristics of MDS Patients

The present study enrolled 66 patients diagnosed with MDS (median age of 74 years (range: 22–89), 60.1% (*n* = 40) females and 39.9% (*n* = 26) males) at the time of diagnosis. Table 1 shows the biodemographic and clinical characteristics of MDS participants. MDS patients were diagnosed according to WHO classification (2016). Nine (13.6%) were diagnosed with MDS-SLD, 10 (15.2%) with MDS-RS, 40 (60.6%) with MDS-MD, and 7 (10.6%) with MDS-EB. The IPSS-R prognostic score was lower in 38 (57.6%) patients, higher in 13 (19.7%), and not reported in 15 (22.7%). In 15 MDS patients, cytogenetic abnormalities were detected, with 37 patients good, 11 intermediate, and 2 poor cytogenetic profiles. One patient had *FLT3* internal tandem duplication (ITD) mutation, and no patient had TET2 mutations. Of these patients, 44 (66.7%) received erythropoiesis-stimulating agent treatment as supportive care, 7 (10.6%) received azacytidine treatment, and 5 (7.6%) received hydroxyurea. The control group consisted of 26 subjects without hematological, oncological, or oxidative-stress-related diseases (median age of 67 years (range: 32–79), 53.8% females (*n* = 14) and 46.2% males (*n* = 12)).

### 3.2. Oxidative Stress Levels in MDS Patients

In order to analyze the participation of oxidative stress in MDS development, we examined the systemic levels of reactive oxygen and nitrogen species (peroxides and NO), antioxidant defenses (uric acid, vitamin E (plasmatic and erythrocytic), vitamin A, GSH, TAS, erythrocytic GPX and GR activities), and macromolecule oxidative damage (8-OHdG and MDA (plasmatic and erythrocytic)) between patients and controls. Moreover, since oxidative stress results from the imbalance between free radicals and antioxidants levels, we also calculated the peroxide/TAS and NO/TAS ratios. 

**Figure 1:**
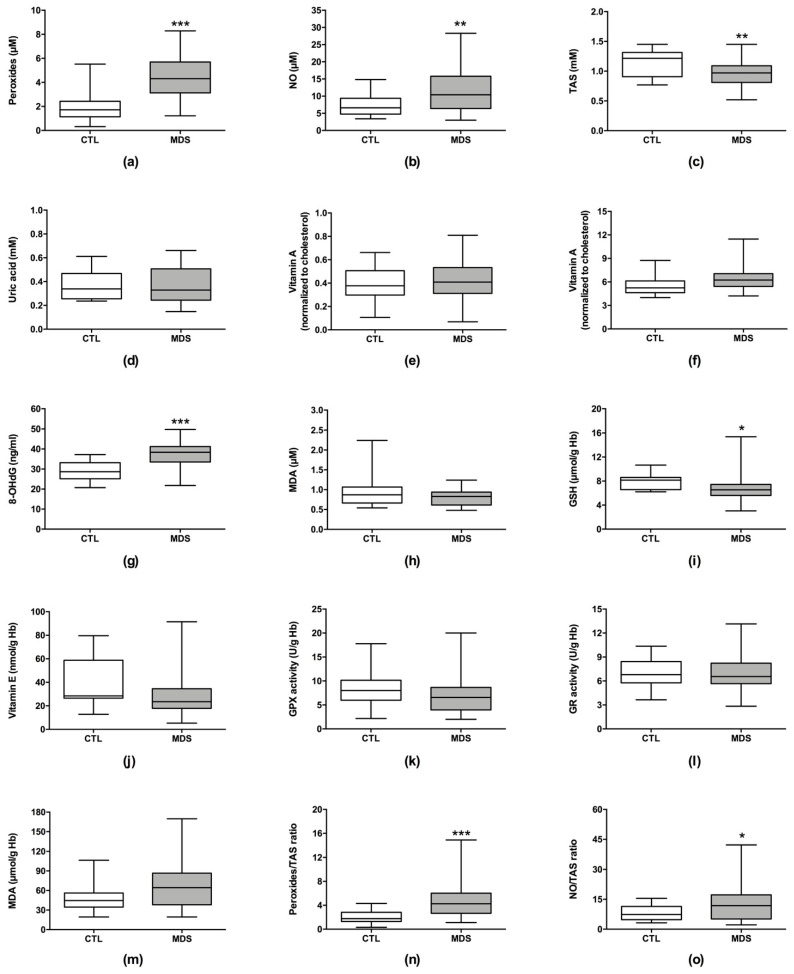
Analysis of plasmatic and erythrocyte oxidative stress parameters in myelodysplastic syndrome patients and controls. The following plasmatic oxidative stress levels are represented: (**a**) peroxides, (**b**) nitric oxide (NO), (**c**) total antioxidant status (TAS), (**d**) uric acid, (**e**) vitamin A, (**f**) vitamin E, (**g**) 8-hydroxy-2-deoxyguanosine (8-OHdG), (**h**) malondialdehyde (MDA), (**n**) peroxide/TAS ratio, and (**o**) NO/TAS ratio. Additionally, the following erythrocyte oxidative stress levels are represented: (**i**) reduced glutathione (GSH), (**j**) vitamin E, (**k**) glutathione peroxidase (GPX) enzymatic activity, (**l**) glutathione reductase (GR) enzymatic activity, and (**m**) MDA. *, *p* < 0.050; **, *p* < 0.010; ***, *p* < 0.001.

As shown in Figure 1, several plasmatic oxidative stress parameters were increased in MDS patients. These patients have higher levels of peroxides (4.32 µM (3.13–5.69), *p* < 0.001), NO (10.40 µM (6.41–15.80), *p* = 0.002), 8-OHdG (38.40 ng/mL (33.50–41.21), *p* < 0.001), peroxide/TAS ratio (4.25 (2.65–6.03), *p* < 0.001), and NO/TAS ratio (11.84 (5.01–17.23), *p* < 0.021) in comparison with controls (peroxides: 1.72 µM (1.14–2.43); NO: 6.60 µM (4.78–9.38); 8-OHdG: 28.68 ng/mL (25.16–33.18); peroxide/TAS ratio: 1.75 (1.30–2.83); NO/TAS ratio: 7.39 (4.76–11.45)). MDS patients also have lower levels of plasmatic TAS (0.97 mM (0.81–1.09), *p* = 0.005) and erythrocytic GSH (6.54 µmol/g Hb (5.61–7.44), *p* = 0.022) than controls (TAS: 1.22 mM (0.91–1.32); GSH: 8.16 µmol/g Hb (6.58–8.61)). Additionally, the MDS-MD subtype showed 2.0- and 2.1-fold higher levels of peroxides and peroxide/TAS ratio in comparison with MDS-SLD (*p* < 0.05) (Table 2). No significant differences were observed between lower- and higher-risk patients (IPSS-R risk groups).

### 3.3. DNA Methylation Status in MDS Patients

**Figure 2:**
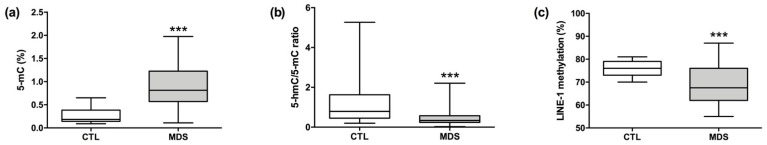
Global and localized DNA methylation statuses in myelodysplastic syndrome (MDS) patients and controls (CTL). Global methylation was determined by the quantification of: (**a**) 5-methylcytosine (5-mC), (**b**) 5-hydroxymethylcytosine (5-hmC)/5-mC ratio, and (**c**) long interspersed nuclear element-1 (LINE-1) methylation. ***, *p* < 0.001.

The DNA methylation status was measured through global DNA methylation (5-mC, 5-hmC/5-mC ratio, and LINE-1 methylation levels; Figure 2). The levels of 5-mC were increased in patients with MDS (0.81% (0.60–1.23); *p* < 0.001), while the 5-hmC/5-mC ratio (0.34 (0.24–0.57); *p* < 0.001) and the LINE-1 methylation (68% (62–76); *p* < 0.001) were decreased when compared with controls (5-mC: 0.18% (0.14–0.38); 5-hmC/5-mC ratio: 0.79 (0.45–1.63); LINE-1: 76% (73–79)). In regard to localized DNA methylation (Table 3), MDS patients had a significant higher methylation frequency of the P15 (48.5%, 32/66), DAPK (42.4%, 28/66), and KEAP1 (33.3%, 22/66) gene promoters when compared with controls (P15: 11.5%, 3/26; DAPK: 0.0%, 0/26; KEAP1: 11.5%, 3/26). Furthermore, the majority of MDS patients had at least one methylated gene (65.5%, 43/66) and, to a lesser extent, two or more methylated genes (48.5%, 32/66). Controls did not have two or more methylated genes. No methylation of the TP53 and MGMT gene promoters was detected in MDS patients and controls. 

### 3.4. Oxidative Stress and DNA Methylation as MDS Diagnostic Biomarkers

Logistic regression was performed to assess whether oxidative stress and DNA methylation factors were associated with MDS development (Table 4). From the several parameters associated with MDS in the univariate analysis, only the levels of peroxides (OR: 1.31, 95% CI: 1.23–1.45, *p* = 0.008), 8-OHdG (OR: 1.23, 95% CI: 1.01–1.59, *p* = 0.041), and 5-mC (OR: 2.13, 95% CI: 1.30–3.48, *p* = 0.003) and the presence of two or more methylated genes (OR: 4.52, 95% CI: 3.39–8.80, *p* < 0.001) were independent predictors of MDS development in the multivariate logistic regression. To analyze the diagnostic value of the independent predictors found in the multivariate logistic regression, we constructed ROC curves (Table 5). The levels of peroxides (AUC = 0.877, 95% IC: 0.800–0.955, *p* < 0.001) and 8-OHdG (0.863, 95% IC: 0.790–0.937, *p* < 0.001) were accurate biomarkers to discriminate MDS patients from controls. The cut-off values were projected to be 3.28 µM and 34.7 ng/mL, respectively, for peroxide and 8-OHdG levels, which achieved good sensitivity (73% and 72%), specificity (92% and 92%), and predictive values (positive predictive value (PPV): 96% and 96%; negative predictive value (NPV): 77% and 80%). However, 5-mC had the greatest diagnostic potential with the highest AUC (AUC: 0.936, 95% CI: 0.887–0.984, *p* < 0.001). The optimal cut-off value of 5-mC was 0.48%, and this cut-off value achieved good sensitivity (82%), specificity (96%), and predictive values (PPV: 93%; NPV: 78%).

### 3.5. Correlation between Oxidative Stress and DNA Methylation in MDS Patients

**Figure 3:**
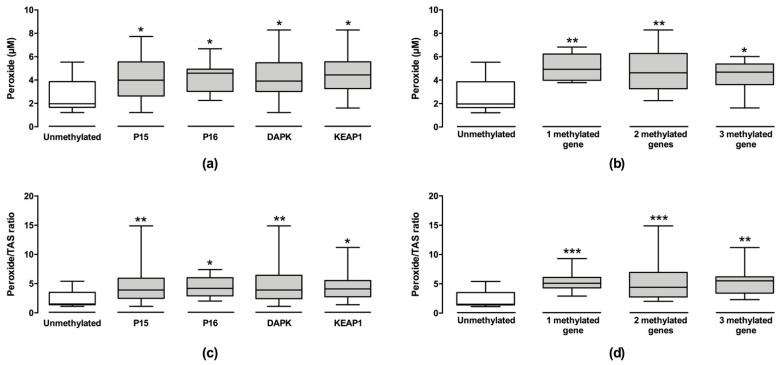
Analysis of oxidative stress parameters in myelodysplastic syndrome patients according to localized DNA methylation status (**a**–**d**). The levels of peroxides (**a**) and peroxides/TSA (**c**) were analyzed in patients stratified according to their methylation statuses of the P15, P16, DAPK, and KEAP1 genes. Next, the levels of peroxides (**b**) and peroxides/TSA (**d**) were examined in patients stratified according to the number of methylated genes. *, *p* < 0.050; **, *p* < 0.010; ***, *p* < 0.001.

To test the hypothesis that oxidative stress levels can be associated with DNA methylation, we analyzed oxidative stress parameters according to P15, P16, DAPK, and KEAP1 gene promoter methylation profiles and correlated the levels of LINE-1, 5-mC, and 5-hmC/5-mC with the oxidative stress parameters. We found that patients with methylated genes had higher levels of peroxides and peroxide/TAS ratio than patients without methylation (Figure 3). The peroxide levels were 2.0-, 2.3-, 2.0-, and 2.3-fold higher in patients with methylated P15, P16, DAPK, and KEAP1 gene promoters (*p* < 0.050), respectively, when compared with patients without methylation. We also observed a significant increase in peroxide/TAS ratio, 2.6- to 2.8-fold higher, in patients with these genes methylated when compared with those without methylation (*p* < 0.050). Moreover, we observed that peroxide levels and peroxide/TAS ratio were significantly increased in patients with one (peroxide: 4.93 µM (3.99–6.23), *p* = 0.002; peroxide/TAS ratio: 5.10 (4.30–6.10), *p* < 0.001), two (peroxide: 4.63 µM (3.27–6.23), *p* = 0.001; peroxide/TAS ratio: 4.40 (2.75–6.95), *p* < 0.001), and three methylated genes (peroxide: 4.69 µM (3.62–5.38), *p* = 0.042; peroxide/TAS ratio: 5.50 (3.40–6.20), *p* = 0.001]) independent of the methylated gene when compared with those patients without gene methylation (peroxide: 1.92 µM (1.66–3.87); peroxide/TAS ratio: 1.50 (1.40–3.50)). 

**Figure 4:**
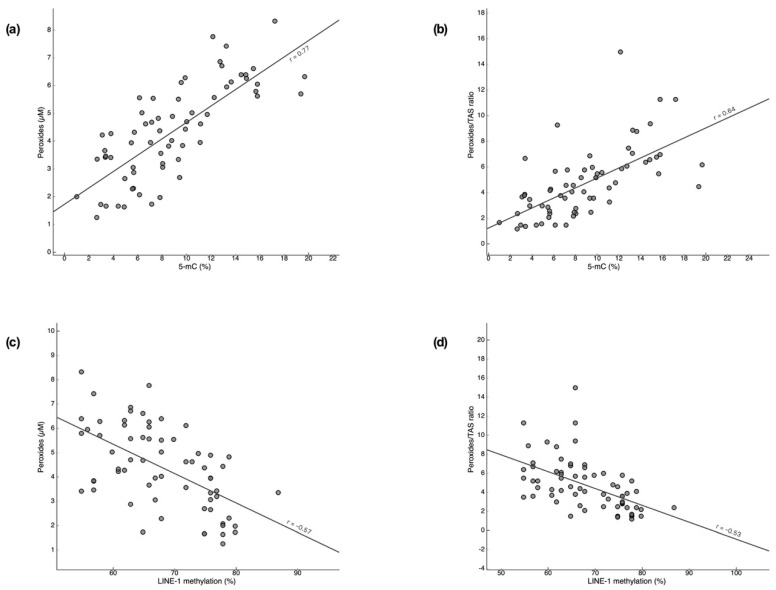
Correlation of oxidative stress parameters with global DNA methylation in myelodysplastic syndrome patients. The following correlations were observed: 5-methylcytosine (5-mC) with peroxides levels (**a**), 5-mC with peroxide/total antioxidant status (TAS) ratio (**b**), long interspersed nuclear element-1 (LINE-1) methylation with peroxides levels (**c**), and LINE-1 with peroxide/TAS ratio (**d**).

Additionally, 5-mC levels were positively correlated with peroxide levels (r = 0.77, *p* < 0.001; Figure 4a) and with peroxide/TAS ratio (r = 0.64, *p* < 0.001; Figure 4b), while LINE-1 methylation was negatively correlated with peroxide levels (r = −0.657, *p* < 0.001; Figure 4c) and with peroxide/TAS ratio (r = −0.53, *p* < 0.001; Figure 4d).

### 3.6. Association of Oxidative Stress and DNA Methylation with MDS Clinical Outcome

**Figure 5:**
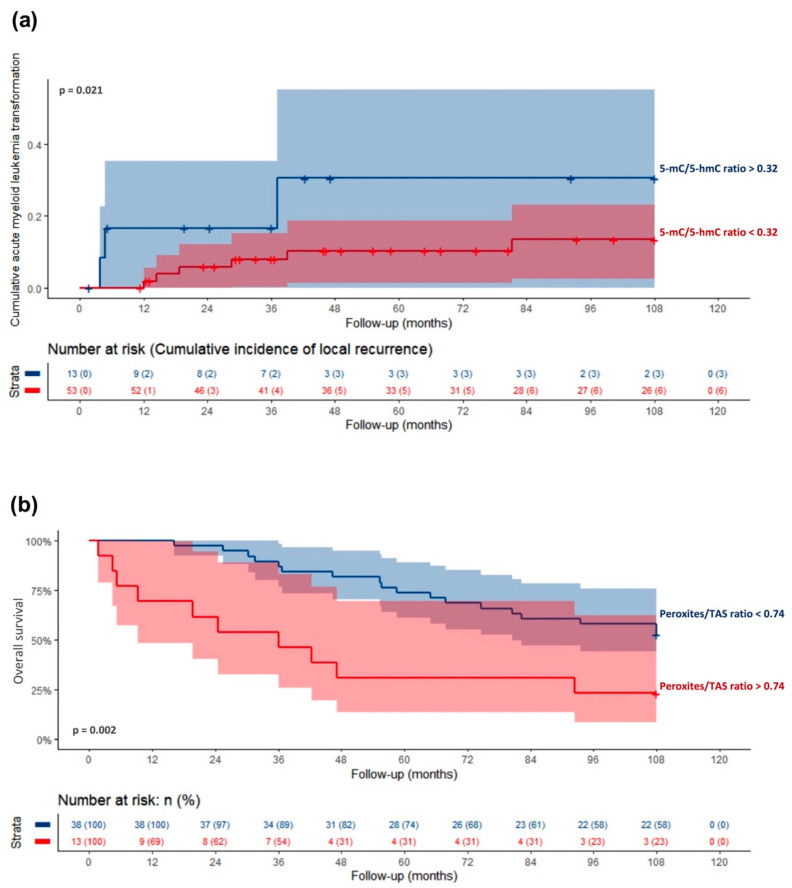
Cumulative acute myeloid leukemia transformation (**a**) and overall survival (**b**) curves of myelodysplastic syndrome patients. MDS patients were stratified through the cut-off points obtained from the ROC curves using a 5-mC/5-hmC ratio for MDS progression and peroxides/TAS ratio for survival.

Nine (14%) patients progressed to AML, and the mean time to progression was 99 ± 4 months. Of the enrolled patients, 38 (58%) died, and the surviving patients had a follow-up time of 108 months, and the mean overall survival of MDS patients was 70 ± 5 months. To determine whether oxidative stress and DNA methylation could influence the progression to AML and the overall survival of MDS patients, we used the ROC analysis. This analysis estimated the best cut-off points that allow us to predict progression to AML and death, and we used them to stratify patients. As observed in Figure 5a, patients with a high 5-mC/5-hmC ratio had a higher progression rate than those with low levels (*p* = 0.012). The mean time to progression of patients with a 5-mC/5-hmC ratio lower than 0.32 was significantly longer (109 ± 3 months) than those with higher levels (87 ± 7 months). On the other hand, patients with a high peroxide/TAS ratio had worse overall survival (Figure 5b). The mean survival time of patients with a peroxide/TAS ratio higher than 0.74 was significantly shorter (59 ± 6 months, *p* = 0.001) than those with lower levels (87 ± 7 months).

## 4. Discussion

Different cancer models suggest that high ROS levels contribute to cancer development and progression through genetic and epigenetic mechanisms. Regarding epigenetic events, ROS can induce both tumor suppressor hypermethylation and global DNA hypomethylation [15]. In the present study, we demonstrated a correlation between DNA methylation and oxidative stress levels in MDS patients. First, we confirmed our previous results showing that hypermethylation of the *P15* and *P16* gene promoters were correlated with ROS levels and with peroxide/TAS ratio. Then, we showed, for the first time, that hypermethylation of the *DAPK* and *KEAP1* gene promoters, 5-mC levels, and LINE-1 methylation were associated with oxidative stress levels (peroxide and peroxide/TAS ratio) in these myeloid neoplasia patients.

Oxidative stress has been implicated in the pathogenesis of several hematological neoplasias, including myelodysplastic syndrome [12,14,44,45,46,47,48,49]. Here, we found that patients with MDS had increased peroxide and nitric oxide levels and decreased levels of GSH and TAS and higher ratios of peroxides/TAS and NO/TAS. These significant disturbances in the balance between free radical and antioxidant levels, in favor of the former, indicate that MDS patients are under oxidative stress [27]. Moreover, and as a consequence of these redox imbalances, MDS patients show oxidative stress damage compared with healthy individuals. In fact, MDS patients showed increased levels of DNA damage, evidenced by an increase in the 8-OHdG levels. These findings were also observed in other studies [28,45,49,50]. Additionally, we found that the involvement of OS in MDS might be dependent on the MDS subtype, once MDS-MD patients had higher levels of peroxides and peroxide/TAS ratio than MDS-SLD patients. These results suggest the involvement of OS in the number of hematopoietic lineages affected and, consequently, could be related to the number of cytopenias observed in these patients. ROS levels modulate cellular signaling pathways, which lead to proliferation or apoptosis in a stress-level-dependent manner [12]. For example, ROS induces extrinsic and intrinsic apoptoses through JNK activation or by decreasing cellular GSH levels that conduce to redox imbalance [51]. This fact may contribute to the increased susceptibility and multilineage cytopenia observed in this MDS subtype. Importantly, we found that peroxide levels and DNA damage were independent risk factors for MDS development. Moreover, 8-OHdG was found to be an accurate diagnostic biomarker of MDS. The confirmation of these results in independent studies will support and highlight the clinical usefulness of these oxidative damage parameters as MDS diagnostic biomarkers. Altogether, these data support that oxidative stress is involved in the pathogenesis of MDS.

Epigenetic abnormalities, such as changes in DNA methylation patterns, are other key players in the development of myeloid malignancies. In the present study, MDS patients had significant hypermethylation of the *P15* and *DAPK* tumor suppressor genes. These results are similar to those reported in the literature [27,28,52,53]. In MDS patients, we also observed significant hypermethylation of the *KEAP1* promoter gene; however, no other study investigated the methylation status of this gene in hematological malignancies. The KEAP1–NRF2 pathway is the primary protective response to oxidative and electrophilic stresses by regulating the expression of cytoprotective genes and has been implicated in cancer progression and resistance to therapy [54]. KEAP1 is the major negative regulator of cellular defenses against ROS by binding to the NRF2 transcription factor, targeting it to degradation in the ubiquitin–proteasome pathway. When KEAP1 is silenced by hypermethylation, NRF2 becomes activated, leading to antioxidant enzyme expression [43] and contributing to MDS progression. Further, here we found that patients with MDS frequently had one or more methylated genes. These results confirm that abnormal tumor suppressor gene hypermethylation is a common event in MDS [27,52,53,55,56]. Additionally, we found that patients with MDS had increased levels of 5-mC and decreased 5-hmC/5-mC ratio and LINE-1 methylation. As mentioned above, epigenetic abnormalities are common events in myeloid malignancies; however, only a few studies have investigated their global methylation statuses. Figueroa et al. (2010), Yamazaki et al. (2012), Bujko et al. (2013), and Sucic et al. (2019) reported that myeloid neoplasia patients had global hypermethylation, whereas Ko et al. (2010) observed global hypomethylation [57,58,59,60,61]. Scopim-Ribeiro et al. (2014) observed a significant downregulation of TET2 (ten-eleven translocation 2) expression in total bone marrow cells from MDS patients [62]. TET2 belongs to a family of methylcytosine dioxygenases, a group of enzymes that oxidize the methyl group of 5-mC to 5-hmC and other methylcytosines, facilitating demethylation [63]. This fact could contribute, at least in part, to the decreased ratio of 5-hmC/5-mC observed here. Since repeated DNA sequences, such as LINE-1, are enriched in CpG sites, it has been considered that global hypomethylation largely arises from the demethylation of these sequences [64]. Therefore, since a decrease in LINE-1 methylation was observed in our cohort of MDS patients, these patients showed global hypomethylation. This methylation pattern has been associated with genome instability, changes in chromatin structure, and increased frequency of copy number abnormalities [65], also found in MDS patients. To the best of our knowledge, only one study has investigated LINE-1 methylation in MDS patients. In this study, Römermann et al. (2008) reported that LINE-1 was hypermethylated in these patients [66]. However, Bolatti et al. (2009) found that LINE-1 was hypomethylated in multiple myeloma, and Fabris et al. (2011) observed similar results in chronic myelogenous leukemia [67,68]. Despite controversial results in global DNA methylation, the high levels of 5-mC found here could be a molecular explanation for the clinical success of hypomethylating agents, such as decitabine and azacytidine, in these diseases. Despite the success of hypomethylating agents, approximately 50% of patients fail to respond to these drugs [69]. The majority of patients that respond to hypomethylating agents eventually relapse due to the development of primary and secondary resistances to this treatment [69,70]. Moreover, the aberrant DNA methylation pattern observed in the present study reinforces the role of epigenetics in MDS development.

DNA hypomethylation and hypermethylation can be induced by ROS [15]. The positive correlation of LINE-1 hypomethylation and oxidative stress, observed in our cohort of myeloid neoplasia patients, has already been demonstrated in patients with bladder cancer [21]. Several molecular mechanisms could explain the correlation of LINE-1 hypomethylation with oxidative stress. First, cells with increased production of ROS, such as neoplastic cells, require high GSH levels. In this context, cells under oxidative stress may redirect S-adenosylmethionine (SAM)—the universal endogenous donor of methyl groups—to the methionine cycle in one-carbon metabolism to synthesize GSH, resulting in a decreased availability of SAM to DNA methylation processes [71]. Additionally, the increased levels of 8-OHdG may explain the hypomethylation of LINE-1 observed in patients with MDS. The formation of this oxidized DNA base in a CpG site not only may inhibit the methylation of the adjacent cytosine by DNA methyltransferases but can also induce guanine-to-thymine transversion, which will result in the loss of a CpG site [16,21,72]. Additionally, we found a positive correlation of 5-mC and tumor suppressor gene hypermethylation with oxidative stress (peroxide levels and peroxide/TSA ratio). Interestingly, the peroxide levels and the peroxide/TSA ratio were similar between patients with one, two, or three methylated genes. This fact suggests that hypermethylation of tumor suppressor genes is not proportional to oxidative stress levels. The oxidative DNA damage leading to single-strand breaks (SSBs) could be responsible for the increased levels of 5-mC and the hypermethylation of tumor suppressor genes observed in the present study. The incomplete repair of SSBs by base excision repair enzymes results in their conversion in double-strand breaks, signaling for de novo methylation and, therefore, contributing to unprogrammed methylation [73,74]. Furthermore, ROS can induce hypermethylation of tumor suppressor genes by the upregulation of DNMT1 and histone deacetylase 1, enzymes involved in gene silencing through promoter methylation and histone deacetylation [75], and by the formation and relocalization of a silencing complex, composed of DNMT1, DNMT3B, SIRT1, and members of polycomb repressive complex 4, stimulating cancer-specific hypermethylation [76]. Finally, the superoxide anion may directly deprotonate cytosine at the carbon 5 position, allowing the formation of methylated cytosine through the nucleophilic attack of SAM [17]. 

In the present study, some issues must be taken into account. First, although we had enrolled almost all newly diagnosed patients during the recruitment period, this study analyzed a relatively small cohort of patients. This limitation did not allow us to evaluate oxidative stress and methylation parameters in all MDS subtypes. Second, and despite the correlations found between PB and BM in a small number of MDS patients and controls, the same studies must be replicated in isolated neoplastic cells from bone marrow. However, in clinical practice, frequent BM sampling in older people, such as MDS patients, has been associated with significant morbidity. Thus, several studies have compared the dysplastic features of cells and mutation patterns and have found a high concordance of genomic and cytogenetic aberrations between PB and BM in MDS. In this context, since PB cells are more accessible than BM ones, the investigation in PB cells may also be one of the work strengths since PB is a more accessible and less invasive biological sample. Another strength of the study is the employment of robust analytical methods to assess oxidative stress and identify new prognostic biomarkers in MDS.

## 5. Conclusions

The present findings and our previous preliminary study [27] support our hypothesis that DNA methylation is correlated with oxidative stress in MDS patients. We found an association of peroxide levels and peroxide/TAS ratio not only with tumor suppressor gene hypermethylation but also with LINE-1 hypomethylation. Moreover, we showed that hypermethylation of the *KEAP1* gene promoter is a frequent event in MDS patients. Overall, this study reflects the complexity of MDS and points to a possible link between oxidative stress and DNA methylation, two common pathogenic mechanisms of MDS, which also contribute to AML progression and survival and show clinical potential as new prognostic biomarkers in MDS.

## Figures and Tables

**Table 1:** Biodemographic and clinical characteristics of MDS patients.

Characteristics	MDS (*n* = 66)		Controls (*n* = 26)	
Demographic Features				
Gender (%)				
Male	26	(39.9%)	12	(46.2%)
Female	40	(60.1%)	14	(53.8%)
Age (years)				
Median	74		67	
Range	22–89		32–79	
Clinical features				
Hematological parameters (median, range)				
WBC (×10^9^/L)	3.5	(1.3–13.0)		
Hb (g/L)	10.6	(5.4–16.0)		
Platelets (×10^9^/L)	98	(12–324)		
WHO 2016 classification				
MDS-SLD (%)	9	(13.6)		
MDS-RS (%)	10	(15.2)		
MDS-MD (%)	40	(60.6)		
MDS-EB (%)	7	(10.6)		
IPSS-R risk groups				
Lower-risk (IPSS-R score ≤3.5)	38			
Higher-risk (IPSS-R score >3.5)	13			
Not recorded	15			
Cytogenetics				
Good	37			
Intermediate	11			
Poor	2			
Not recorded	15			

MDS, myelodysplastic syndrome; WHO, World Health Organization; WBC, white blood cells; Hb, hemoglobin; MDS-SLD, MDS with single lineage dysplasia; MDS-MD, MDS with multilineage dysplasia; MDS-RS, MDS with ring sideroblasts; MDS-EB, MDS with excess blasts; IPSS-R, Revised International Prognostic Scoring System.

**Table 2:** Plasma levels of peroxides and peroxide/TAS ratio on myelodysplastic syndrome subtypes.

Parameter	MDS-SLD Median (Q1–Q3)	MDS-RS Median (Q1–Q3)	MDS-MD Median (Q1–Q3)	MDS-EB Median (Q1–Q3)	*p*-Value
Peroxides (µM)	2.27 (1.70–4.02)	4.59 (3.03–5.85)	4.54 (3.48–6.07)	4.34 (2.62–5.92)	0.048
Peroxide/TAS ratio	2.30 (1.40–4.15)	4.40 (2.65–5.78)	4.75 (3.50–6.68)	4.50 (2.40–6.50)	0.036

Q, quartile; MDS-SLD, myelodysplastic syndrome with single lineage dysplasia; MDS-MD, MDS with multilineage dysplasia; MDS-RS, MDS with ring sideroblasts; MDS-EB, MDS with excess blasts; TAS, total antioxidant status.

**Table 3:** Methylation frequency of studied genes on myelodysplastic syndrome patients, their subtypes, and controls.

Condition	Methylation Frequency							
	*P15*		*P16*		*DAPK*		*KEAP1*	
	*n*	%	*n*	%	*n*	%	*n*	%
MDS	32/66	48.5 **	13/66	19.7	28/66	42.4 ***	26/66	33.3 *
MDS-SLD	3/9	33.3	3/9	11.1	3/9	33.3	1/9	11.1
MDS-RS	4/10	40.0	4/10	10.0	7/10	70.0	2/10	20.0
MDS-MD	20/40	50.0	20/40	20.0	14/40	35.0	17/40	42.5
MDS-EB	5/7	71.4	3/7	42.9	4/7	57.1	2/7	28.6
Controls	3/26	11.5	1/26	3.8	0/26	0.0	3/26	11.5
	*TP53*		*MGMT*		1 ≥ methylated genes		2 ≥ methylated genes	
	*n*	%	*n*	%	*n*	%	*n*	%
MDS	0/66	0.0	0/66	0.0	43/66	65.2 ***	32/66	48.5 ***
MDS-SLD	0/9	0.0	0/9	0.0	4/9	44.4	1/9	11.1
MDS-RS	0/10	0.0	0/10	0.0	5/10	70.0	3/10	30.0
MDS-MD	0/40	0.0	0/40	0.0	29/40	72.5	23/40	57.5
MDS-EB	0/7	0.0	0/7	0.0	5/7	71.4	5/7	71.4
Controls	0/26	0.0	0/26	0.0	4/26	15.4	0/26	0.0

*, *p* < 0.050; **, *p* < 0.010; ***, *p* < 0.010; MDS-SLD, MDS with single lineage dysplasia; MDS-MD, MDS with multilineage dysplasia; MDS-RS, MDS with ring sideroblasts; MDS-EB, MDS with excess blasts.

**Table 4:** Univariate and multivariate logistic regression analysis in patients with myelodysplastic syndrome.

Biomarkers	Univariate Analysis			Multivariate Analysis		
	OR	95% CI	*p*-Value	OR	95% CI	*p*-Value
						
Peroxide	3.36	1.96–5.76	<0.001	1.31	1.23–1.45	0.008
8-OHdG	1.27	1.43–1.41	<0.001	1.23	1.01–1.59	0.041
Peroxide/TAS	2.54	1.61–3.99	<0.001	–		
NO	1.18	1.05–1.31	0.004	–		
NO/TAS	1.11	1.02–1.21	0.017	–		
5-mC	2.43	1.62–3.64	<0.001	2.13	1.30–3.48	0.003
5-hmC	1.54	1.14–2.09	0.005	–		
LINE-1	0.83	0.75–0.91	<0.001	–		
Methylated *p15*	8.42	3.85–9.40	<0.001	–		
Methylated *KEAP1*	3.83	1.04–7.18	0.044	–		
2 ≥ methylated genes	7.51	4.59–9.34	<0.001	4.52	3.39–8.80	<0.001

OR, odds ratio; CI, confidence interval; 8-OHdG, 8-hydroxy-2-deoxyguanosine; LINE-1, long interspersed nuclear element-1; 5-mC, 5-methylcytosine; 5-hmC, 5-hydroxymethylcytosine; NO, nitric oxide; TAS, total antioxidant status.

**Table 5:** Significant oxidative stress and DNA methylation parameters as a diagnostic biomarker of myelodysplastic syndrome.

Biomarkers	AUC		Cut-Off				
	Value (95% CI)	*p*-Value	Value	SEN (%)	SPE (%)	PPV (%)	NPV (%)
Peroxide (µM)	0.877 (0.800–0.955)	<0.001	3.28	73	92	96	77
8-OHdG (ng/mL)	0.863 (0.790–0.937)	<0.001	34.7	72	92	96	80
5-mC (%)	0.936 (0.887–0.984)	<0.001	0.48	82	96	93	78

OR, odds ratio; CI, confidence interval; 5-mC, 5-methylcytosine; 8-OHdG, 8-hydroxy-2-deoxyguanosine; MDA, malondialdehyde; AUC, area under the curve; CI, confidence interval; SEN, sensitivity; SPE, specificity; PPV, positive predictive value; NPV, negative predictive value.

## Data Availability

All data generated or analyzed during this study are included in this published article.
